# Iconicity in Word Learning and Beyond: A Critical Review

**DOI:** 10.1177/0023830920914339

**Published:** 2020-04-20

**Authors:** Alan KS Nielsen, Mark Dingemanse

**Affiliations:** Max Planck Institute for Psycholinguistics, Netherlands; Centre for Language Studies, Radboud University, Netherlands

**Keywords:** Iconicity, learning, communication, comprehension, bootstrapping

## Abstract

Interest in iconicity (the resemblance-based mapping between aspects of form and meaning*)* is in the midst of a resurgence, and a prominent focus in the field has been the possible role of iconicity in language learning. Here we critically review theory and empirical findings in this domain. We distinguish local learning enhancement (where the iconicity of certain lexical items influences the learning of those items) and general learning enhancement (where the iconicity of certain lexical items influences the later learning of non-iconic items or systems). We find that evidence for local learning enhancement is quite strong, though not as clear cut as it is often described and based on a limited sample of languages. Despite common claims about broader facilitatory effects of iconicity on learning, we find that current evidence for general learning enhancement is lacking. We suggest a number of productive avenues for future research and specify what types of evidence would be required to show a role for iconicity in general learning enhancement. We also review evidence for functions of iconicity beyond word learning: iconicity enhances comprehension by providing complementary representations, supports communication about sensory imagery, and expresses affective meanings. Even if learning benefits may be modest or cross-linguistically varied, on balance, iconicity emerges as a vital aspect of language.

## 1 Introduction

Over 150 years ago, Dwight Whitney, one of the most prominent linguists of the time, declared that “Every existing form of human speech is a body of arbitrary and conventional signs for thought, handed down by tradition from one generation to another” (Whitney, 1867, p. 32). At a stroke, he established what would become a central tenet of linguistics—the idea that words are arbitrary—and connected it to the process of social transmission and therefore language learning. While some had stressed the potential of iconic relations between sound and sense (von Humboldt, 2000 [1836]), the notion of iconicity receded into the background, except for some short cyclical bursts of interest.

**Figure 1.**
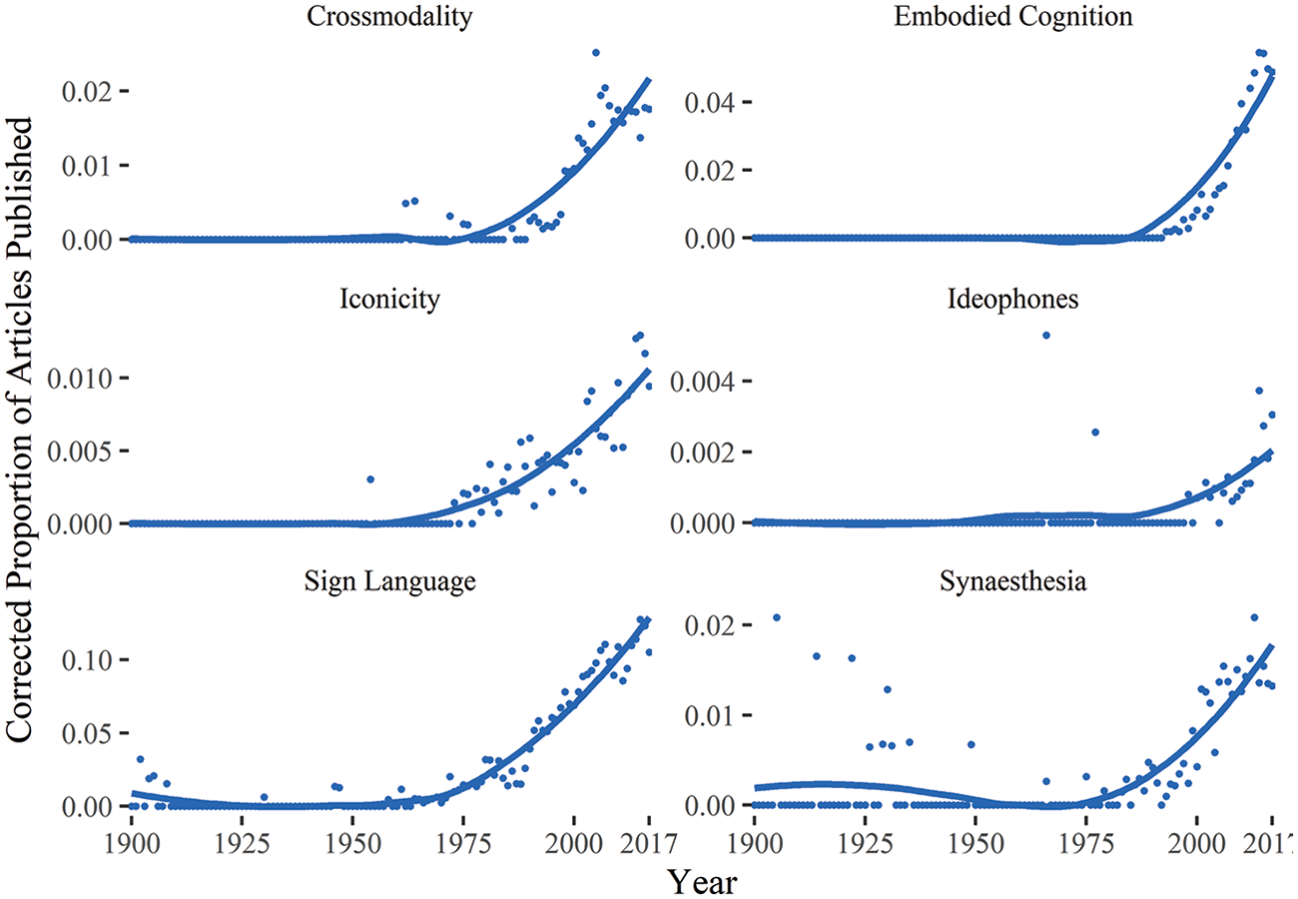
Proportional number of publications cataloged in Web of Science (1900–2017), showing concurrent upsurges in topics related to iconicity (corrected for overall publication volume).

The current surge of interest in iconicity research, however, seems much more sustained and widespread than in previous decades (Figure 1), and for the first time, it is questioning not just the dogma of arbitrariness but also articulating ways in which iconicity may provide practical utility in language learning and communication (Dingemanse et al., 2015; Imai et al., 2008; Perniss & Vigliocco, 2014; Yoshida, 2004).

The renewed interest in iconicity can be traced to a number of factors: the cognitive sciences have ramped up the study of synesthesia and crossmodality (Ramachandran & Hubbard, 2001; Spence, 2011), psychology has become increasingly interested in embodied cognition and depiction (Clark, 2016; Zlatev, 2007), and in linguistics there has been growing attention to lexical iconicity in sign languages and in ideophones, vivid iconic words found in many spoken languages (Svantesson, 2017; Vigliocco & Kita, 2006). This fast-growing, interdisciplinary research area has matured enough for there to be several broad-ranging reviews of the significance of iconicity (Perniss & Vigliocco, 2014; Svantesson, 2017) as well as meta-analyses of experimental work (Fort et al., 2018; Motamedi et al., 2019; Styles & Gawne, 2017). However, with theories and methods still very much in flux, a critical look at some of the key claims is timely.

One of the strongest cases for the utility of iconicity has been made in the domain of word learning (Imai et al., 2008; Maurer et al., 2006). Iconic words are among the earliest learned words, and they are also easier to learn than some other words. Perhaps the perceptual analogies on display in iconic words provide learners with a stepping stone to the principle that words can refer to things in the world, which may subsequently be generalized to other, less iconic types of words (Gogate & Hollich, 2010; Harnad, 2007). On this account, iconicity may provide an initial step out of the symbol grounding problem (Harnad, 1990), though this does not fully resolve the paradox of how to learn other, less iconic words. As we show below, a central open question in this literature is about the extent of the iconic learning advantage: is it local, limited to iconic words, or more general, also scaffolding the learning of less iconic words?

Although we review a range of accounts of the relation between iconicity and word learning, we devote special attention to a proposal that has the twin virtues of being formulated clearly and generating a wide following: Imai and Kita’s (2014) “Sound-symbolism bootstrapping hypothesis.” One of our goals is to assess the state of the evidence for sound-symbolic bootstrapping, generally understood as the notion that iconicity may give the learner a foothold for learning not just iconic words, but also other aspects of language. We suggest that: (a) the strongest evidence for an effect of iconicity on learning deals with local learning benefits; (b) in comparison, the evidence for general learning benefits is relatively meager, and so, (c) claims about the importance of iconicity for early language learning (as opposed to word learning) should be tempered.

While we focus primarily on the bootstrapping literature, a crucial argument we make is that the findings in this domain are best understood in the context of a broader take on iconicity: one that recognizes forms of iconicity beyond sound symbolism and functions of iconicity beyond word learning, including enhancing comprehension, supporting communication, and expressing affective meaning. For this broader take on iconicity we draw on work from a range of fields, including communication, gesture studies, linguistics, and sign languages (Ortega, 2017; Perniss et al., 2010; Tversky, 2011; Valenzeno et al., 2003). One relevant line of work in this area focuses on how the learning of spoken words can be facilitated by iconic co-speech gestures that supply redundant imagistic information (Kelly et al., 2009; Kelly et al., 2017). This work shows local learning benefits and makes no claims about bootstrapping effects, but is nonetheless a good example of how iconicity, by supplying imagistic information, can help in word learning and communication.

### 1.1 Definitions

Iconicity in the vocal domain is often referred to as sound symbolism, although the idea that speech sounds can be mapped onto meanings in motivated ways has gone by a number of names historically. The term *phonetic symbolism* came into common use in psychology and linguistics following seminal work by Sapir (1929) and Newman (1933), but has gradually been overtaken by *sound symbolism*. Though we focus mainly on spoken words (see Ortega (2017) for the view from sign language), we prefer to use *iconicity* as an overarching term, defined as “the resemblance-based mapping between aspects of form and meaning” (Dingemanse et al., 2015). Using *iconicity* connects us to a broader literature on signals that may support iconic associations. So onomatopoeic words like “cluck” are iconic, but so too are words in which reduplication evokes repetition, like the Japanese ideophone *gorogoro* “a heavy object rolling repeatedly” (Vigliocco & Kita, 2006). While linguistic work in this area has tended to focus on forms of iconicity attested in natural languages, experimental work has often used artificial stimuli like *bouba* and *kiki*. A well-known and relatively robust result in this area is that people tend to map the former to “rounded” shapes and the latter to “jagged” shapes (Styles & Gawne, 2017), something that has been connected to a range of possible form–meaning resemblances, from auditory perceptual qualities (Nielsen & Rendall, 2011) to orthography (Cuskley et al., 2015).

Iconicity is distinct from *systematicity*, when statistical regularities in word forms correlate with aspects of word function (Dingemanse et al., 2015; Nielsen, 2016). The critical distinction is that for iconicity, form–meaning associations are based on resemblance, whereas for systematicity, any statistical regularity will do. For example, English nouns contain significantly more syllables than verbs (cp. *boomerang, smirk*), and probably because of this, English speakers are more likely to treat long nonwords as nouns and monosyllables as verbs (Cassidy & Kelly, 1991). Such cues, while usually too subtle to reach conscious awareness, may help language users and learners to assign words to grammatical categories (Kelly, 1992). Note that there is nothing inherently multisyllabic about noun meanings, or monosyllabic about verb meanings; so, this is not a case of iconicity but of systematicity, as seen by the fact that it could have easily been the reverse and speakers would still be able to pick up the regularity. Because systematicity and iconicity are orthogonal dimensions, it is possible for a given word to be related to its meaning both iconically and systematically (Nielsen, 2016). In this review we are concerned mainly with iconicity, but we will flag the distinction where relevant.

## 2 Iconicity and Learning

Early work on iconicity in language rarely considered the possibility that iconic associations between words and meanings might be important for learning. For psychologists, sound symbolism offered a useful illustration of cross-sensory correspondences, with implications for the nature of analogy in perception (Köhler, 1947; von Hornbostel, 1927). For linguists, sound symbolism was a curiosity found to varying degrees across languages, mostly in sensory vocabulary (Jespersen, 1922; Sapir, 1929; Westermann, 1927). Thus most of this work focused not on learning but on exploring biases in sound–meaning associations found experimentally and in natural languages.

Following a first spate of exploratory research, Taylor and Taylor (1965) introduced a useful distinction between *subjective phonetic symbolism* (“association felt by individuals between individual sounds and their symbolic connotations in arbitrary contexts”) and *objective phonetic symbolism* (“frequent occurrence of individual sounds in words of certain concepts in natural languages”). They also proposed there was feedback between the two: given a language with objective phonetic symbolism, its users will develop subjective symbolism, and given a population with subjective symbolism, the language that population speaks will eventually develop objective symbolism.

Taylor and Taylor’s work suggested caution in jumping to conclusions based on subjective associations alone: if experimental participants associate high-front vowels like /i/ with small objects and low back vowels like /ɑ/ with large objects, and their language also contains objective phonetic symbolism in the same direction (e.g., with oppositions like huge, humongous, enormous vs. tiny, wee, little), how can we say whether the bias shaped language or language was shaped by the bias (cf. Atzet & Gerard, 1965; Jespersen, 1922; Maltzman et al., 1956)? It also pointed to the importance of cross-linguistic data: if a non-arbitrary pattern is found in multiple unrelated populations or languages, this increases the chance that it is grounded in more widespread cognitive or perceptual biases, as opposed to being merely an example of language-specific systematicity or a result of shared history.

### 2.1 A Recent Upsurge

Where early work focused on identifying iconic patterns (in languages) and iconic biases (in language users), the possibility that iconicity might itself influence language learning was only teased at. Recently, however, the suggestion that iconicity can enhance learning has enjoyed growing interest: the proportion of publications on the intersection of iconicity and learning cataloged in Web of Science has increased seven-fold since 2001.

A number of papers stand out as influential. Ramachandran and Hubbard (2001) connected synesthesia and sound symbolism in a general synesthetic bootstrapping theory of language origins. Their suggestion that iconicity (underpinned by shared synesthetic cross-modal associations) can help explain how people learn to connect form and meaning has been echoed numerous times (Cuskley & Kirby, 2013; Nielsen & Rendall, 2011); see Table 1. Gasser (2004) demonstrated with a computational model that non-arbitrary mappings between forms and meanings are both easier to learn and easier to store. Learning studies showed that iconicity has advantages for word learning (Yoshida, 2004): for 3-year-old Japanese children, newly created iconic words for manner of motion were easier to learn than non-iconic ones (Imai et al., 2008), and similar iconic mappings in existing Japanese words facilitated word learning for English adult speakers (Nygaard et al., 2009). In a relatively short period of time, iconicity moved from being considered a marginal aspect of language to a potent force in language learning (Asano et al., 2015; Perniss & Vigliocco, 2014; Thompson et al., 2012).

**Table 1.** Key statements on the role of iconicity in language learning.

Study	Statement about the role of iconicity in bootstrapping
Ramachandran and Hubbard (2001)	“The key idea here is that each of these different effects (synaesthesia between object appearance and sound contour, between sound contour and vocalizations, and synkinaesia) in isolation may have been too small to have exerted adequate selection pressure for the emergence of proto-language, but a bootstrapping between all of them acting together may have indeed been sufficient.”
Maurer et al. (2006)	“the bouba/kiki phenomenon exists early enough in development that it can indeed facilitate the learning of languages in which rounder objects tend to be labelled with rounded vowels” (p. 321)
Imai et al. (2008)	“[s]ound symbolism facilitates one of the most important tasks for children in language development, namely, the learning of novel verbs.” (p. 63) “Its investigation leads to the important question of how iconicity in multi-sensory mappings bootstraps children to break the initial barrier for language learning” (p. 63)
Gogate & Hollich (2010)	“[redundancy across modalities] scaffolds early learning of auditory-visual relations and provides a foundation for the learning of sound-symbolic relations. . . . Only over time, as a critical mass of sound-symbolic relations accrue, is it possible to learn more arbitrary relations” (p. 506)
Thompson (2011)	“The presence of iconicity across languages and its role in language acquisition suggests an additional mechanism whereby iconicity provides scaffolding (a middle ground) to bridge the ‘great divide’ between linguistic form and human experience”
Monaghan, Shillcock, Christiansen, & Kirby (2014)	“The corpus analyses we have conducted are entirely consistent with views that sound symbolism may be necessary for bootstrapping word learning.”
Perniss & Vigliocco (2015)	“Here, we propose that iconicity provides an additional, critical mechanism for reducing referential ambiguity and therefore for promoting word/sign learning.”
Bankieris & Simner (2015)	“. . .cross-modal mechanisms that are explicit in synaesthetes may indeed aid them when understanding sound symbolic words, and we suggest this may have facilitated comprehension in early states of vocabulary development in language evolution.”

### 2.2 Bootstraps, Bridges, and Scaffolds

Claims that iconicity can make language learning easier often use metaphors from the broader literature on language development: bootstrapping, bridging, and scaffolding. The most common of these is bootstrapping, which tends to refer to a process by which learners use relatively simple initial cues to break into a more complex system.

However, what exactly iconicity is hypothesized to bootstrap, for whom, and by what mechanism, differs from study to study (Table 1). Depending on the account, iconic bootstrapping may help in the emergence of proto-language, the learning of iconic words, the subsequent learning of more arbitrary words, or even language learning in general; and its beneficiaries may be language users in general, subgroups like children or synesthetes, or all of the above.

One of the most fully laid out theories of the relation between iconicity and learning is Imai and Kita’s (2014) *sound symbolism bootstrapping hypothesis* (cf. Imai et al., 2008). Its key claim is that “sound symbolism helps children learn the meaning of words at different stages of early lexical development” (p. 4). Despite being named analogously to semantic bootstrapping (Pinker, 1996 [1984]) and syntactic bootstrapping (Gleitman, 1990)—both capturing simple processes that enable learners to generalize beyond the input—the sound symbolism bootstrapping hypothesis is not so much a single process as a convenient shorthand for a collection of observed and hypothesized learning advantages connected to iconicity. These advantages are laid out by Imai and Kita in the form of five claims (p. 4):

**P1** “Children, even pre-verbal infants, are sensitive to sound symbolism. . .”
**P2** “Young children are sensitive to a wider range of possible sound-symbolic associations than adults. . .”
**P3** “Sound symbolism helps infants who have just started word learning to gain the insight that speech sounds refer to entities in the world. . .”
**P4** “Sound symbolism helps infants associate speech sounds and their referents. . .”
**P5** “Sound symbolism helps toddlers identify referents embedded in a complex scene. . .”

Premise 1 is fairly uncontroversial, though there are conflicting reports about the developmental trajectory of children’s sensitivity to iconicity (as discussed in 3.1 below). Premise 2 seems plausible by analogy with the categorical perception literature, but seems to have no support yet in published work: infant studies have tended to focus on sound–shape and sound–size associations (Fort et al., 2018; Peña et al., 2011), both well attested in adults yet representing only a tiny sliver of the possibility space of iconic mappings (Thompson & Do, 2019).

Premises 3–5 spell out the subsidiary claims relating to bootstrapping most clearly: sound symbolism may help infants gain the insight that speech sounds can refer (i.e., establish symbol grounding; P3), may help infants establish links between particular speech sounds and referents (P4), and may help toddlers identify referents in complex scenes (P5). Let us consider the evidence for these key premises in more detail, starting with the latter two.

There is ample evidence that iconicity facilitates the learning of iconic words and their meanings (P4 and P5): onomatopoeia are among the earliest learned words (Laing, 2019), iconically congruent form–meaning pairs are easier to learn or to retain for young toddlers (Kantartzis et al., 2019; Miyazaki et al., 2013), and sound-symbolic forms for manner-of-motion meanings are more easily generalized to novel instances than arbitrary forms for children as well as adults (Imai et al., 2008; Kantartzis et al., 2011; Yoshida, 2012). On the other hand, the premise that iconic form–meaning mappings can help early learners to discover that speech sounds can have a referential function (P3) seems to have less direct empirical support. The single study cited in support of it is suggestive of sound-symbolic sensitivity in 11-month-olds, but stops short of showing how this might help infants to pick up sound-referent associations that are arbitrary (Asano et al., 2015). More research is needed to establish whether sound symbolism may help infants to gain referential insight.

Despite the empirical prudence of Imai and Kita’s account, which focuses mainly on how iconicity can make iconic words easier to learn, studies citing and summarizing it often extend its claims to the subsequent “learning of non-iconic words” (Massaro & Perlman, 2017, p. 10), to “infant’s word learning” in general (De Carolis et al., 2017), or even more broadly to “language learning” (Perry et al., 2017; Sidhu & Pexman, 2017). It is clear from this that for many scholars, iconic bootstrapping extends far beyond the learning of iconic words. This underscores the need to take a step back and consider what evidence we actually have, and which open questions need to be resolved to clear up the role of iconicity in learning.

### 2.3 Local Versus General Learning Enhancement

How exactly may iconicity help word learning? We start by distinguishing between two types of learning enhancement: *local learning enhancement*, where iconic words are themselves easier to learn, and *general learning enhancement*, where the learning of iconic words influences the subsequent learnability of less iconic words or other aspects of language. Local learning enhancement could explain why, for example, the highly iconic English word “roar” is early acquired, along with many other words rated as highly iconic (e.g., “crunch,” “beep,” “roar”; Perry et al., 2017). General learning enhancement would suggest that learning such iconic words influences one’s ability to subsequently learn less iconic words (e.g., “hamster,” “silent,” and “pyjamas”) or other features of language.

What does current evidence allow us to conclude about the role of iconicity in language learning, either in terms of local or general learning enhancement? Let us start by formulating some basic observations about iconicity and word learning that are, as far as we can ascertain, entirely uncontroversial:

**I** Iconic words are statistically overrepresented among early-learned words
**II** Iconic words are somewhat easier to learn than arbitrary words
**III** Eventually, learners acquire a vocabulary that is largely arbitrary

First, we know that in several languages, the early-learned portion of the lexicon is rated as relatively more iconic than later-learned portions (Massaro & Perlman, 2017; Perry et al., 2015; Thompson et al., 2012). Second, we observe that for a variety of reasons, and agnostic to mechanism, iconic words are somewhat easier to learn (this subsumes Imai and Kita’s premises 4 and 5 and the evidence for them discussed above). Using the terminology introduced above, this can be construed as local learning enhancement. Third, we know that nearly everyone who grows up surrounded by language eventually acquires a fully functional language of their own, including large swathes of vocabulary that show more arbitrariness and less iconicity (Bühler, 1934; Gasser, 2004; Harnad, 2007).

Each of these observations is likely to be true in the general sense—the key question is *why* these happen to be facts of word learning. Observation I recognizes that the relationship between iconicity and age of acquisition is statistical, rather than absolute. It thereby leaves room for the possibility that iconicity has its own developmental trajectory (reviewed in 3.1) and that it has functions beyond early word learning (reviewed in 3.4). Observation II invites careful consideration of the strength of local learning benefits (reviewed in 3.2). It also allows for discussion of the mechanisms by which iconic words become easier to learn, which may include facts about cross-modal correspondences or common neural coding (Sidhu & Pexman, 2017) as well as features of the input, such as the fact that in child-directed speech, adults preferentially use iconic words (Perry et al., 2017) and salient prosody (Reinisch et al., 2013). Observation III captures a broad consensus that sufficiently large vocabularies feature larger amounts of arbitrariness, but it does leave room for cross-linguistic differences in the prevalence and distribution of iconic and arbitrary words (reviewed in 3.3).

The three observations above can be combined into a proposal similar to those made in work on iconic bootstrapping:

**IV** The early learning (I) of easy-to-learn iconic words (II) is followed by the eventual acquisition of a sizable arbitrary vocabulary (III)

This observation, stripped of causal language, reminds us that mere sequential order is often strongly suggestive of causality even when such a conclusion may not be warranted (Michotte, 1963). Many interpretations of the sound symbolism bootstrapping hypothesis appear to assume that the early learning of iconic items must, in some way, cause the later-learned less iconic tokens to be easier to acquire. This aligns with our notion of general learning enhancement. But it is only one logical possibility: the eventual acquisition of a fuller vocabulary may be enhanced by, contingent on, or even unrelated to the early learning of iconic words. One of the most urgent tasks in the field of iconicity and learning is to spell out the differences between these scenarios.

If the role of iconicity in learning is limited to local learning enhancement, this would predict the early acquisition of some easier-to-learn words, which would be sufficient to account for the bulk of the learning effects reported so far but may imply traceable cross-linguistic differences. If the role of iconicity extends to general learning enhancement, we must be able to specify how exactly the early learning of iconic words contributes to the subsequent learning of less iconic aspects of language. For instance, we may predict that the proportion of iconic words in the input is a significant predictor of subsequent lexical growth. And if overall learning is fully unrelated to early iconic word learning, this predicts no traceable relation, and also leads to the expectation of diminished cross-linguistic differences in this area. In each case, iconicity clearly plays an important role in word learning, but the different scenarios generate testable predictions about (differences in) learning mechanisms, age of acquisition, vocabulary size, and possible variation in learning outcomes conditioned by cross-linguistic differences.

Part of the reason for our agnostic take on these matters is simply parsimony: we should not seek answers that are more complex than are warranted by the data. But a second source of uncertainty is the fact that the current state of evidence for the first three claims is not always as clear cut as it is characterized in the literature. Below, we review some evidence to this effect, suggesting that challenges of experimental design and a lack of good data on the acquisition and distribution of iconicity across languages challenge a simple causal role of iconicity as a general learning facilitator. Iconicity, we argue, is best thought of as one of multiple possible cues helping word learning; and moreover, a cue that has other functions in language as well. The next sections consider challenges and opportunities in this domain, starting with topics directly related to learning and broadening out to other functions of iconicity which risk being obscured by the field’s focus on word learning.

## 3 Challenges and Opportunities

Above we considered the notion of general learning enhancement while operating under the assumption that our other two premises are indeed correct: iconic words are easier to learn, and are learned earlier, than arbitrary words. However, the actual state of empirical evidence is not entirely straightforward. Here we focus on four areas that present important challenges and opportunities for research on iconicity and learning: the developmental trajectory of iconicity; the experimental study of iconicity; the differential distribution of iconicity; and the utility of iconicity beyond learning.

### 3.1 The Acquisition of Iconicity

Although the greater prevalence of iconic tokens in the early input and early lexicon of children is statistically robust, it is a rather small effect (Perry et al., 2017). Children learn many conventional words in addition to iconic ones, and those words that are iconic vary considerably in their degree of iconicity. So, toddlers do not appear to have sufficient difficulty with acquiring non-iconic tokens as to make that kind of learning impossible early on: iconicity has a notable but nowhere near exhaustive influence on the lexicons of language learners. Indeed, parental checklist data for 1089 children indicates that that by the time they produce their first words, children already understand around 60 words that are not, on average, rated as highly iconic by adults, like *bath, diaper, eat, juice*, and *kiss* (Massaro & Perlman, 2017; cf. also Goodwyn & Acredolo, 1993).

One of the few studies to directly test the relation between the influence of various lexical input features on lexical development found no significant contributions from the occurrence of onomatopoeia or iconic words on subsequent lexical growth in 47 English-learning infants from 9 to 21 months (Ota et al., 2018).The study found that not iconic words, but diminutives and reduplication in the input (whether in iconic or non-iconic words) were associated with vocabulary growth. However, it should be pointed out that in many languages, the types of expressive and evaluative morphology represented by reduplication and diminutives overwhelmingly co-occur with iconic vocabulary (Mattes, 2018), suggesting it may be artificial to dissociate them.

There is also evidence that the ability of children to acquire iconic form–meaning associations has its own developmental trajectory, with a possibly protracted sensitive period (Sourav et al., 2019) and with older children more sensitive to iconicity than young children. This is the case both for the artificial word stimuli typical of sound symbolism studies and for existing iconic words from unfamiliar languages (Tzeng et al., 2017). Notably, the youngest children (3-year -olds) in Tzeng et al.’s study were not sensitive to the sound symbolism of existing words, suggesting that iconicity in the lexicon is not an automatic ticket to understanding. Such results provide reason for caution in interpreting evidence based on pseudowords constructed to show maximally congruent form–meaning associations.

Meanwhile, even maximally sound-symbolic pseudowords do not guarantee success in preverbal infants: 12-month-olds are sensitive to sound/shape correspondences but preverbal 4-month-old infants are not (Pejovic & Molnar, 2017), and a recent meta-analysis of sound symbolism in early language development concludes that “this type of sound symbolism is weak in the earliest stages of life” (Fort et al., 2018). This comprehensive meta-analysis, which pooled data from 425 participants across 11 extant studies (six published and five unpublished), found support for a moderate but significant effect of sound symbolism with several details pointing to the importance of learning in shaping sensitivity to iconicity. Studies with children between 4 and 15 months had smaller effect sizes than with older children; children are more sensitive to bouba-type pseudowords, without an effect of age; and sensitivity to kiki-like pseudowords increased with age. Fitting the trend of increased sensitivity from age 2 and onwards, a recent study found an iconicity advantage in 3-year-olds, where sound-symbolically matching pseudowords were learned and retained better than sound-symbolically mismatching ones (Kantartzis et al., 2019): a clear and important case of iconicity facilitating local learning enhancement.

The literature on iconic gestures provides much the same picture. In studies of symbol learning, recognition of how a gesture resembles its referent was found in 26-month-olds, but not in 18-month-olds (Namy et al., 2004). Similarly, Magid & Pyers (2017) found that 3-year-old children were unable to guess the meaning of iconic gestures at rates above chance, whereas 4-year-olds performed above chance, and were better at guessing the correct meaning for iconic gestures than arbitrary ones. Children also show limited sensitivity to iconic counting gestures, only becoming able to reliably make use of the iconic mapping between counts of fingers and numbers after the age of four (Nicoladis et al., 2018). In short, the recognition of iconicity is not automatic but requires some cognitive and semiotic skills to be already in place.

The combined weight of evidence suggests that the role of iconicity in word learning may be more complicated than supposed: if the processing and understanding of iconicity has its own developmental trajectory and occurs partly in parallel with non-iconic word learning rather than prior to it, iconicity loses some of its bootstrapping appeal, and it becomes more critical to understand the distribution and functions of iconicity by itself. Future work should focus on exploring the acquisition trajectories of more and less iconic aspects of language, with special consideration to seeking out causal explanations by combining learning experiments with rich developmental corpora.

Finding causal evidence is crucial for making claims about the function of iconicity for learning, especially when we consider Taylor and Taylor’s (1965) point about objective versus subjective sound symbolism: some words in the early-acquired portion of the lexicon might be rated as more highly iconic by adult speakers simply because they were learned early and so gave rise to subjective feelings that the word-meaning mapping is iconic (Jespersen, 1922).

### 3.2 Experimental Challenges

Experiments designed to catalog and quantify iconic associations between words and meanings have come a long way in the past century (Motamedi et al., 2019). Experimenters have introduced standardized creation of stimuli according to well-described procedures (e.g., Monaghan et al., 2012) and/or normed by naive participants in pre-tests (e.g., Sidhu & Pexman, 2016). Increased rigor has clarified some earlier findings, with careful manipulations of stimuli revealing that the influence of iconic associations on learning or matching is not exceptionally strong (Fort et al., 2015; Nielsen & Rendall, 2011) and that iconicity is only one among multiple factors influencing learnability.

One such factor concerns systematicity. In many cases, associations between words and meanings are systematic in addition to being iconic. For example, the *kiki*-like words from Maurer et al. (2006) all contain voiceless plosive phonemes (“kuh-tay,” “kay-kee,” “tee-tay,” “tuh-kee-tee”) while the bouba-like words all contain voiced (sometimes sonorant) consonants (“baa-moo,” “boo-baa,” “go-gaa,” “maa-boo-maa”) (Styles & Gawne, 2017). In designs like this, it is possible for experimental participants to respond in line with purportedly iconic biases based on systematicity—that is, the recognition that there are effectively two sets of words that overlap minimally in their features and two sets of images with the same quality. The fact that participants generally adhere to the iconic systematic mapping in their responding, rather than a counter-iconic one, does suggest that iconicity is still important, but the influence of systematicity on their responses cannot be overlooked. Nielsen et al. (in revision) found that fully systematic sets of words were easier to learn across the board, whether those words were iconic in addition to being systematic, conventionally (i.e., non-iconically) systematic, and surprisingly even when the languages to be learned were counter-iconic (see also Brand et al., 2017).

Research on existing iconic words like ideophones has provided further support for the importance of task demands and experimental transparency on the strength of putatively iconic associations. In typical experimental set-ups, participants are presented with a single iconic word and asked to choose the best fitting meaning out of two possible translations, one of which corresponds to the original meaning and one of which is a distractor (Lockwood et al., 2016). When participants are able to choose from antonym pairs, their ability to guess the meanings of ideophones from unfamiliar languages is reliably above chance. However, even in these cases iconicity does not provide a very large advantage, and so it should perhaps be unsurprising that with a wider array of choices, and in the absence of existing semantic knowledge or contextual cues, word-guessing experiments become much more difficult (Brackbill & Little, 1957). For instance, attempting to guess which of two Czech words has the same meaning as a Japanese ideophone (Maltzman et al., 1956), or guessing the meaning of ideophones that have their prosodic or phonological representations shifted (Dingemanse et al., 2016) is considerably harder for participants. That such tasks are harder, or that learning biases may be comparatively small, is not in itself an argument against the utility of iconicity in (local) learning enhancement: after all, given the socially transmitted nature of words, even small biases will ensure a selective advantage over generations of learners (Tamariz & Kirby, 2015; Thompson et al., 2016).

Collectively, these challenges point toward the need for better documentation and standardization of practices and protocols in the field, including the use of shared sets of experimental stimuli that will allow the results of disparate studies to be more easily compared with each other. There are further opportunities available for researchers who study ideophones, including moving beyond the typically studied Japanese ideophone system and studying the role of prosody and gesture. Crucially, such methodological improvements might allow the field to make more general claims about the signatures and functions of iconicity in natural languages.

### 3.3 The Distribution of Iconicity

Are all learners exposed to the same sorts of iconicity, or is the ability to recognize the iconic nature of words conditioned on the features of one’s language? Some iconic biases appear to be fairly strong—for example, many of the world’s languages use reduplicated forms for meanings in the domain of movement and plurality (Mattes, 2018). But if individual learners are biased toward these kinds of associations, why don’t all languages show them? Clearly, other forces are at play when it comes to the distribution of iconicity both within and between languages: language change (Flaksman, 2017; Nuckolls, 1999), the affordances of different modalities and language systems (Little et al., 2017; Perlman et al., 2018), competition between different iconic mappings (Gordon & Heath, 1998; Thompson & Nielsen, 2017), and the need to retain sufficient expressive flexibility (Monaghan et al., 2011) are all forces that shape and constrain the emergence and maintenance of iconicity in the lexicon (Dingemanse et al., 2015).

Corpus studies of the early acquisition of iconic words show a high prevalence of onomatopoeia like *baa, meow, choo-choo*, and *woof* (Akita, 2009, pp. 27–32; Laing, 2014; Massaro & Perlman, 2017; Menn & Vihman, 2011). In a study of German diary data, Laing (2014) showed that early-acquired onomatopoeia (e.g., *meow*) tolerate a large degree of articulatory freedom, and make way for corresponding arbitrary forms (*cat*) when the child’s phonological system is sufficiently developed. So, onomatopoeia provide the infant with a fall-back option to achieve communicative goals in the absence of full phonological capabilities. Here we already get a sense of the importance of iconicity for areas beyond word learning: it provides an early communicative advantage. Onomatopoeia are universally available in spoken languages, which increases the prospects that any advantages they offer in early communicative development are of universal relevance. Indeed Laing (2019) notes that several features of onomatopoeia—including salient forms, consonant harmony, open syllables and reduplication—have clear learning and production advantages.

However, to the extent that languages differ in the prevalence, distribution, and linguistic status of iconic words (Nuckolls, 1999; Svantesson, 2017), there may be consequences for theories of learning and iconicity. Importantly, the main results underlying the sound-symbolism bootstrapping hypothesis are based on iconic manner-of-motion pseudowords patterned after ideophones (Imai et al., 2008), a lexical class that is well developed in Japanese but not to the same extent in all languages (Dingemanse, 2019). Cross-linguistic differences in the distribution of iconic words are reflected in normalized MCDI data: the Japanese MCDI inventory includes about three times as many iconic words as the English one, and many of these ideophones depict event structure, not just sound (Yoshida, 2004). So it appears Japanese children have early access to a higher proportion of iconic words.

This could be a crucial test case for adjudicating between local versus general learning enhancement: do Japanese children just learn a larger number of iconic words, or are there knock-on effects for their concurrent or subsequent early verb learning? Do children learning languages like English or German, with comparatively smaller stocks of iconic words, have fewer opportunities to benefit from iconic cues in word learning? In general, cross-linguistic differences bring home the importance of global linguistic diversity to address questions about the role of iconicity in word learning and beyond.

### 3.4 Functions of Iconicity

An implicit assumption in virtually all of the experimental work reviewed here is that the main function of iconicity is to facilitate learning. However, words are learned because they provide some utility, and in everyday language use, iconic words serve a wide range of functions. To understand the affordances of iconic words, it is useful to zoom out to the supercategory of iconic signs, which includes ideophones, iconic gestures, and visualizations. Iconic signs are external representations that present structural correspondences between domains by means of resemblances (Larkin & Simon, 1987). Because they bear a resemblance to what they depict, they can convey some content and structural relations directly. Because they are selective in what they depict (they are more like diagrams than replicas), they support schematization and abstraction (Tversky, 2011). This combination of analogy and abstraction makes them powerful communicative and cognitive tools.

The power of iconicity is well known from research on iconic co-speech gestures. Such gestures have been found to enhance comprehension (Chu & Kita, 2008; Kelly et al., 2010) by highlighting perceptual-motor information (Alibali & Kita, 2010; Alibali et al., 2011) and by supplying imagistic information harder to encode in arbitrary words (Holler et al., 2009; Rowbotham et al., 2014).

Ideophones are in many ways the closest spoken analog to manual gestures (Kunene, 2001). Japanese has many ideophones iconically conveying aspects of pain sensations, which are widely used in medical interaction and provide patients and doctors with iconic, gradient ways to express sensations that are otherwise hard to verbalize (Hamano, 1998; Sakamoto et al., 2014). Japanese and English speakers converge on how they associate at least some phonological cues in these words to the temporal profile and intensity of pain sensations, confirming their iconic properties (Iwasaki et al., 2007). Texture ideophones, found in many languages, provide a similar case: they iconically encode salient psychophysical dimensions of haptic touch, and are common in episodes of collaborative learning and manual labor, where participants hone in on haptic properties of food being processed or crops being harvested (Mihas, 2013; Nakagawa, 2013). Across languages, ideophones are also often used in storytelling, where they vividly depict manners and paths of motion in ways that complement more prosaic verbal resources (Ibarretxe-Antuñano, 2004; Kita, 1997; Nuckolls, 2014). Further, ideophone-like vocal depictions are common in workplace meetings, dance classes, and music lessons, where participants use them to calibrate understanding and bodily behavior (Clark, 2016; Keevallik, 2014).

Experimental work on the use of iconic signals in interaction confirms the communicative utility of iconicity. For instance, people can successfully link vocal depictions to the bodily movements of dancers, probably relying on shared cross-modal correspondences and perceptual analogies (Styles, 2016). Work in experimental semiotics show that iconicity plays an important role in the initial negotiation of novel communicative conventions (Fay et al., 2014; Garrod et al., 2007; Scott-Phillips et al., 2009). A study of the cultural evolution of artificial languages found an increase in iconicity over generations when participants had to coordinate on meanings, but not when they merely had to learn and reproduce meanings. This suggests that iconic signals provide a selective advantage in cultural transmission primarily because they help people achieve mutual understanding (Tamariz et al., 2018).

These examples from diverse communicative contexts are united in two key ways. First, all of them represent typical “adult” uses of iconic signs: the focus is not on learning the words but on using them for their iconic affordances. Second, they all show how iconicity can enhance comprehension in context by offering representations that are complementary to arbitrary or propositional formats (Campisi & Özyürek, 2013). Whether in the context of doctor–patient interaction, storytelling, or experimental semiotics, iconic signs are highly effective tools for communicating imagistic, perceptually grounded information.

Besides their communicative utility, iconic signs are also commonly used for aesthetic and expressive effect. Words rated as highly iconic but acquired late, like *shrill, gnarled*, and *rustle* (Perry et al., 2017), are notable for their poetic and affective connotations. Similarly, words rated as highly iconic are often also rated as high in arousal, as in *jolt, roar*, and *gush* (Warriner et al., 2013). Poets and playwrights are experts at playing with the iconic affordances of speech sounds (Jakobson & Waugh, 1979; Nuckolls, 1999; Samarin, 1970). The esthetic use of iconic words relies in part on the same properties that make them useful as communicative tools. Perceptually grounded analogies and abstractions guide the imagination of the listener and help conjure up poetic imagery. Additionally, the esthetic use of iconic words is supported by expressive and affective associations at the phonological and prosodic level (Aryani et al., 2016; Dingemanse and Thompson, 2020; Klamer, 2002).

Humans are not the only animal to take advantage of iconic or otherwise motivated associations between the forms of signals and their meanings. Animal communication systems broadly have a pressure for *functional deployability*—the ability of signals to “do work” for the signaler (Krebs & Dawkins, 1984; Nielsen & Rendall, 2018). In some cases, this work involves the transfer of propositional content (information), while in other cases the work to be done refers to modulating the behavior of others (manipulation). Thus, calls made in aggressive contexts are often harsh, loud, and broadband in nature, while those made in affiliative contexts are relatively softer and more mellifluous —as in a cat hissing aggressively versus one purring contentedly (Morton, 1977). These types of signals are not semantic in the sense that human words are, but nonetheless the form of the signal is fitted to its purpose. Human communication also bears the fingerprints of this pressure, both in non-speech vocalizations and in the structure of actual words. Curse words and swears are a case in point: words like “fuck” are highly evocative and can be functionally leveraged and analogically modified for a variety of purposes (Potts, 2007). These words are performative not just because of their conventional meanings, but also by virtue of their prosodic features and phonological make-up: they show rather than tell (Wharton, 2003).

In short, while iconicity can help word learning, the actual uses of iconicity go far beyond this. Recognizing the communicative, esthetic, and expressive functions of iconicity helps solve a paradox. If we assumed, for the sake of argument, that the main role of iconicity was to bootstrap language learning, we would expect it to be relevant mostly in early language development, and we would expect inventories of iconic words to be limited to the repertoire found in child-directed language. We might even expect iconic words to be acquired, leveraged to gain expertise in language generally, and then discarded by adult speakers. But this is not what we find—iconic words extend far beyond simple onomatopeia in meaning and use (Diffloth, 1972; Nuckolls, 1999; Perniss et al., 2010), and in many languages ideophones make up a lexical class on the same order of magnitude as nouns and verbs (Dingemanse, 2019). To understand the prevalence and distribution of iconic words in natural languages, we need to look beyond learning.

## 4 Conclusion: From Magic Bullet to Multiple Affordances

Sustained empirical work on diverse languages has shown that iconicity is more prevalent in the lexicon than assumed. We have reviewed evidence and arguments for the role of iconicity in language learning. There is robust evidence for local learning enhancement: for a variety of reasons, iconic words are easier to learn, which helps explain their relative overrepresentation in early vocabulary. Adults might preferentially direct iconic words to children because those are the words that children are likely to understand. The current state of evidence for general learning enhancement is less robust: it is not clear at present that learning iconic words facilitates the subsequent learning of non-iconic elements of language.

The overrepresentation of iconic words in early vocabulary has sometimes been taken to imply that the learning of less iconic words is contingent on or enhanced by knowing iconic words; however, as we have argued, sequential order does not imply causality, and most current evidence points to local rather than general learning effects. Rather than a magic bullet that can explain the mystery of phonological development or early verb learning, iconicity emerges as one of many cues in a multifaceted learning problem, with a developmental trajectory of its own and with effects that may be subject to cross-linguistic differences. All of these factors make the relationship between iconicity and learning an exciting area for future research. The work reviewed here allows us to draw up three elements of a research agenda for work on iconicity and learning:

*I. Diversify and share stimulus materials beyond bouba and kiki.* A wider range of stimulus materials is needed to systematically assess the potential of iconic form–meaning mappings. Open materials will boost cumulative progress.
*II. Use linguistic diversity as a natural laboratory.* To test causal proposals, compare learning trajectories and acquisition rates across modalities, or in languages with and without sizable classes of iconic words.
*III. Triangulate methods.* Combine observational and experimental evidence to formulate causal models of iconicity in word learning and beyond.

Theories of iconic bootstrapping are an important step in the direction of understanding one possible role of iconicity in language, but as a field we should be mindful of other affordances of iconicity, lest we make the mistake of searching for our keys only underneath the street lamp. We have reviewed evidence for the formidable power of iconicity in communication, and for its expressive functions in more playful and poetic contexts. The same features that make some iconic words easier to learn also make them highly effective tools for conveying content by means of resemblance and schematization.

Indeed, learning advantages may be seen as a special case of the general cognitive advantages offered by analogical representation: thus iconic words might be more learnable in part because they enhance comprehension, are more indexical in their relationship to internal states of both signalers and receivers, and simply more fun. At the extreme, one might propose that iconicity having a positive influence on word learning is parasitic on these other functions, although we prefer the more nuanced view that learnability is one of multiple affordances of iconicity in natural language. Whatever the case, the field of iconicity will thrive not by putting all of its eggs into the learning basket, but by systematically charting the forms and functions of iconicity across the spectrum of human communicative competence—from early language development to everyday language use.
